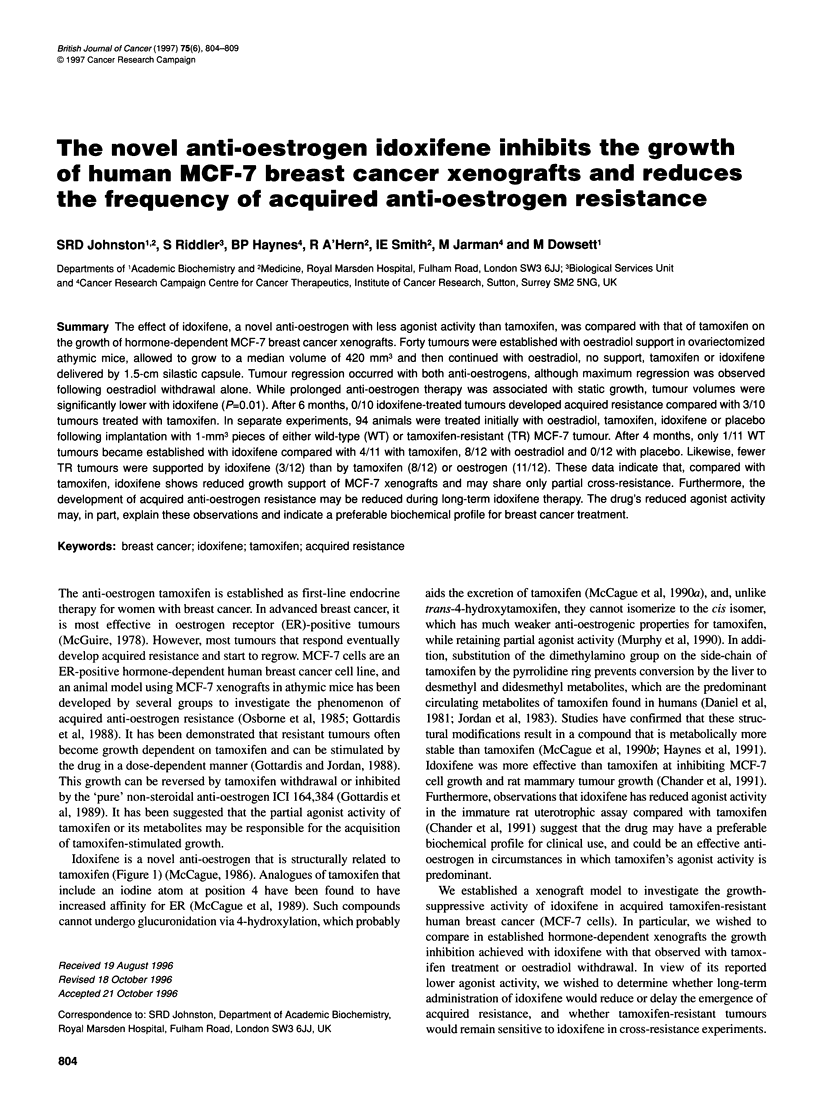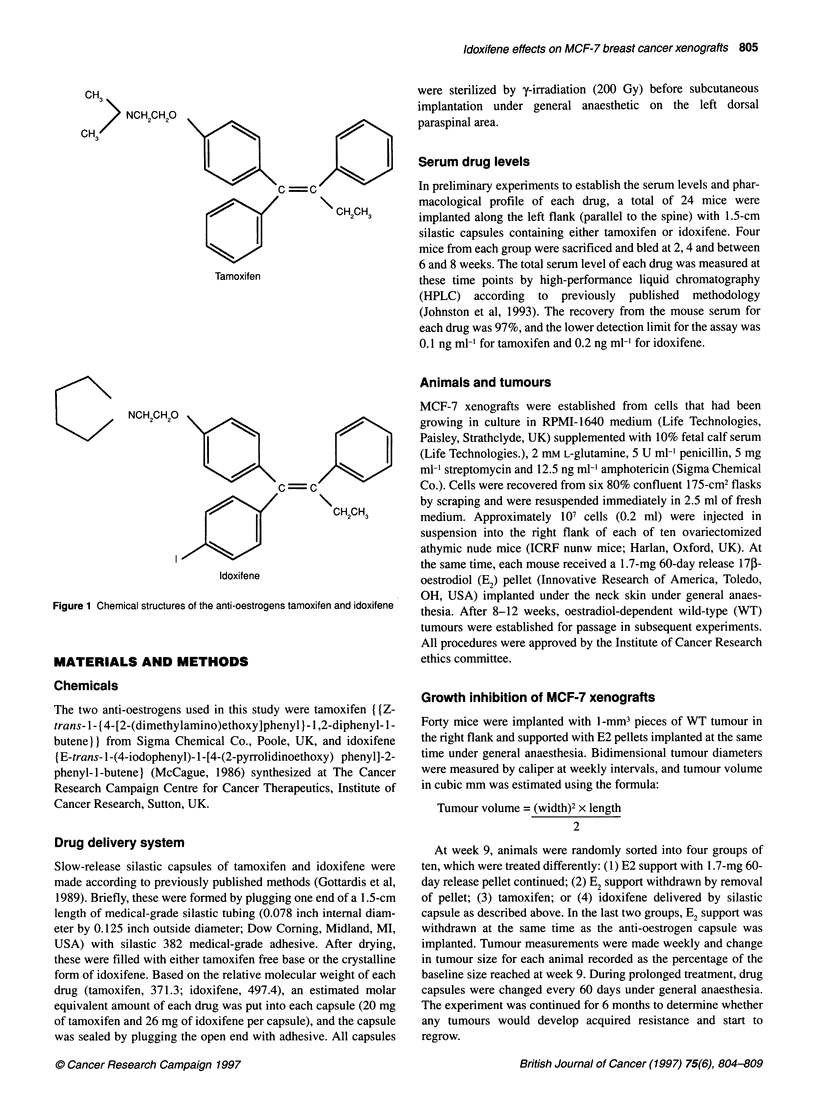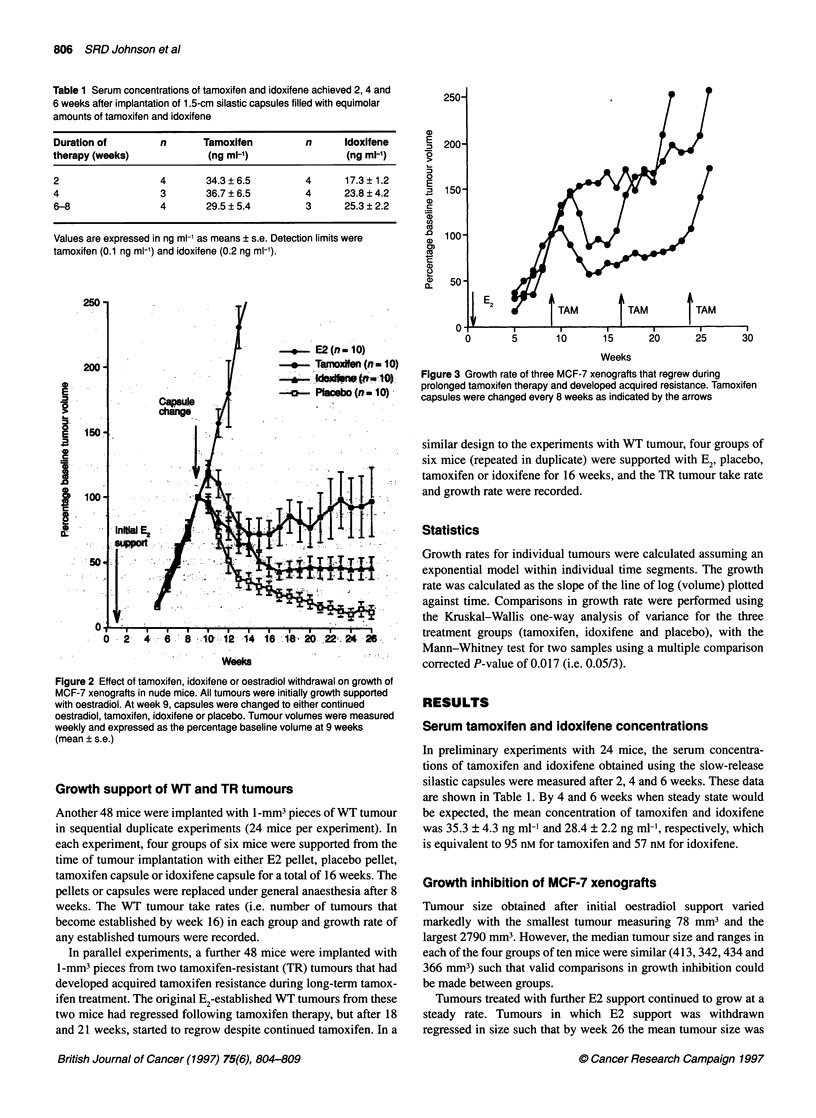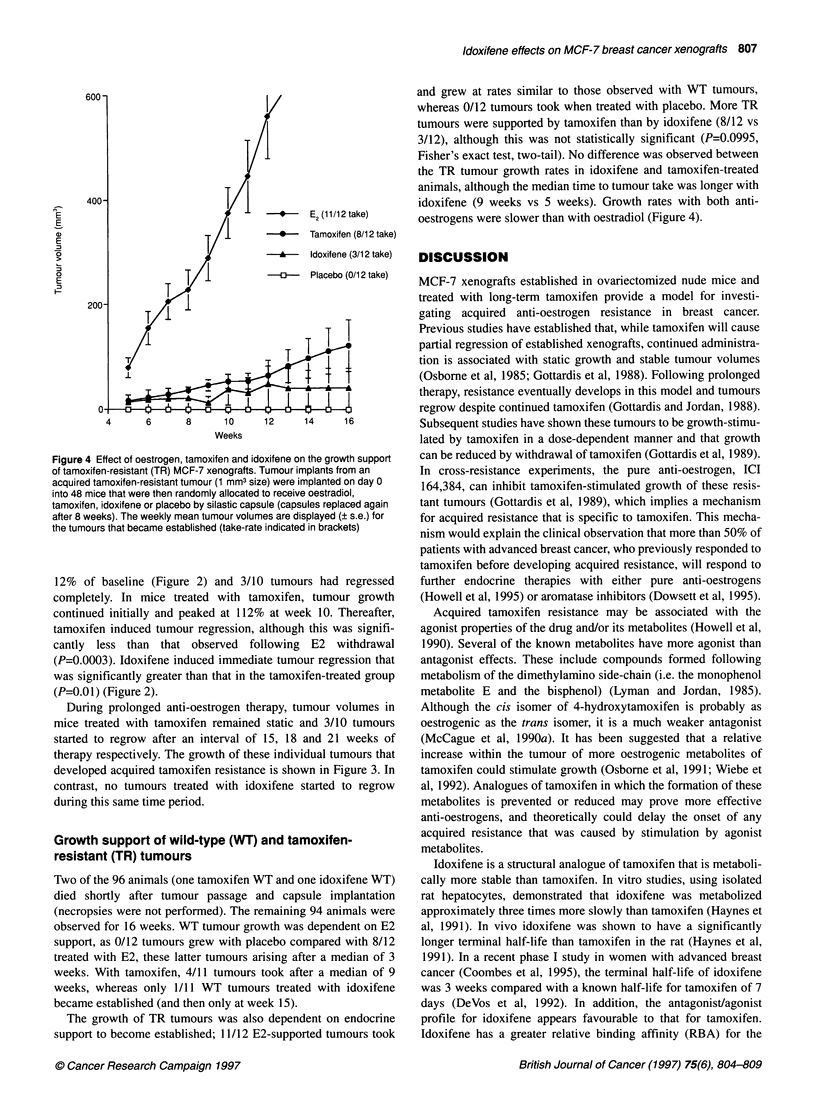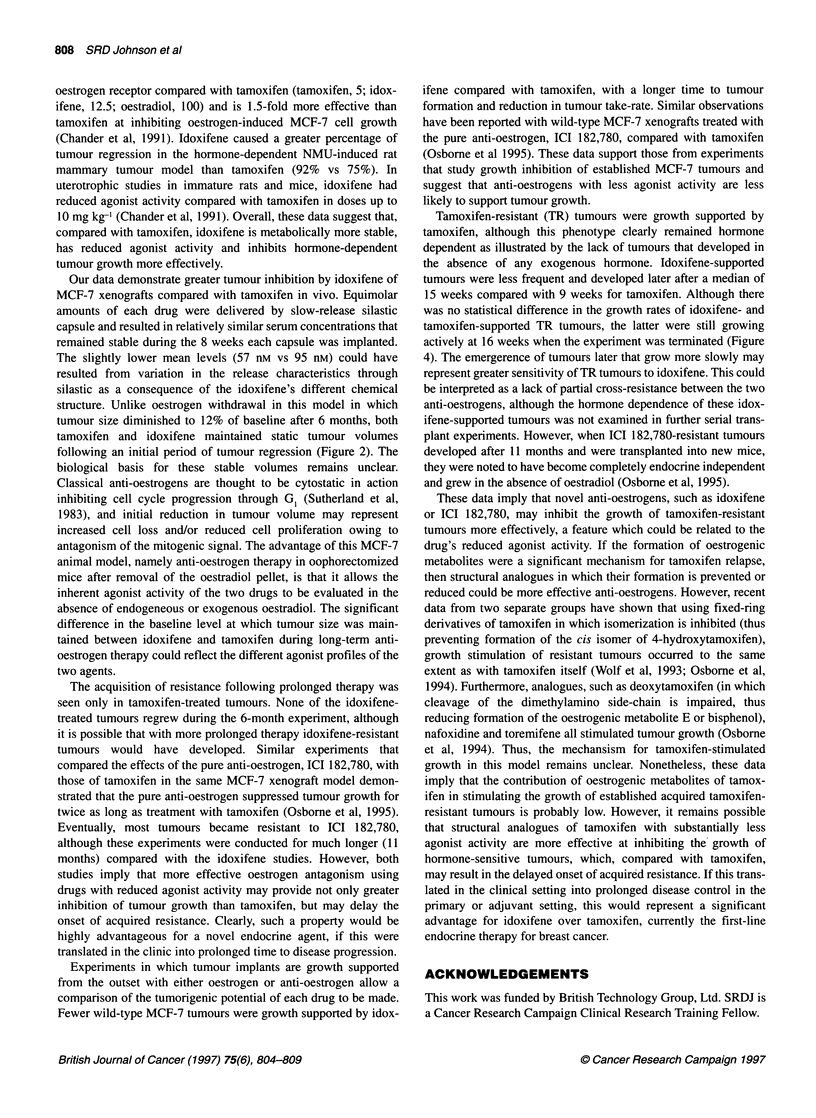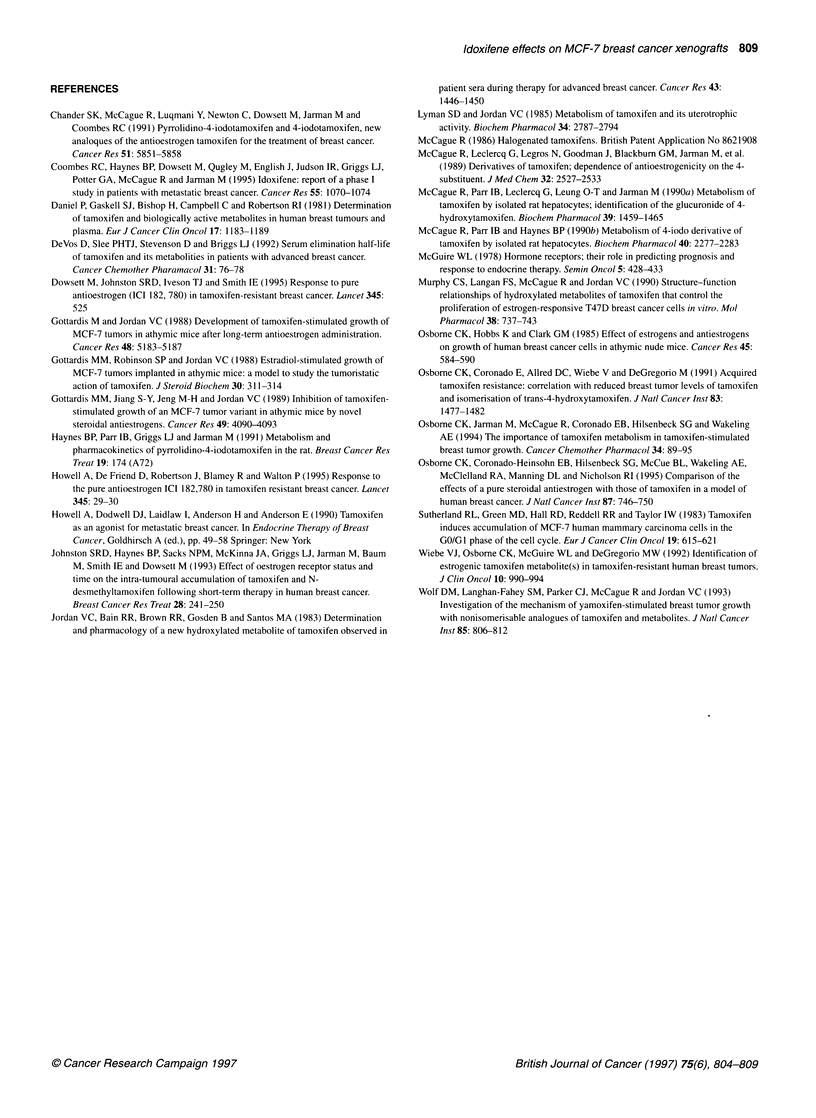# The novel anti-oestrogen idoxifene inhibits the growth of human MCF-7 breast cancer xenografts and reduces the frequency of acquired anti-oestrogen resistance.

**DOI:** 10.1038/bjc.1997.144

**Published:** 1997

**Authors:** S. R. Johnston, S. Riddler, B. P. Haynes, R. A'Hern, I. E. Smith, M. Jarman, M. Dowsett

**Affiliations:** Department of Academic Biochemistry, Royal Marsden Hospital, London, UK.

## Abstract

The effect of idoxifene, a novel anti-oestrogen with less agonist activity than tamoxifen, was compared with that of tamoxifen on the growth of hormone-dependent MCF-7 breast cancer xenografts. Forty tumours were established with oestradiol support in ovariectomized athymic mice, allowed to grow to a median volume of 420 mm3 and then continued with oestradiol, no support, tamoxifen or idoxifene delivered by 1.5-cm silastic capsule. Tumour regression occurred with both anti-oestrogens, although maximum regression was observed following oestradiol withdrawal alone. While prolonged anti-oestrogen therapy was associated with static growth, tumour volumes were significantly lower with idoxifene (P=0.01). After 6 months, 0/10 idoxifene-treated tumours developed acquired resistance compared with 3/10 tumours treated with tamoxifen. In separate experiments, 94 animals were treated initially with oestradiol, tamoxifen, idoxifene or placebo following implantation with 1-mm3 pieces of either wild-type (WT) or tamoxifen-resistant (TR) MCF-7 tumour. After 4 months, only 1/11 WT tumours became established with idoxifene compared with 4/11 with tamoxifen, 8/12 with oestradiol and 0/12 with placebo. Likewise, fewer TR tumours were supported by idoxifene (3/12) than by tamoxifen (8/12) or oestrogen (11/12). These data indicate that, compared with tamoxifen, idoxifene shows reduced growth support of MCF-7 xenografts and may share only partial cross-resistance. Furthermore, the development of acquired anti-oestrogen resistance may be reduced during long-term idoxifene therapy. The drug's reduced agonist activity may, in part, explain these observations and indicate a preferable biochemical profile for breast cancer treatment.


British Journal of Cancer (1997) 75(6), 804–809
© 1997 Cancer Research Campaign

### The novel anti-oestrogen idoxifene inhibits the growth of human MCF-7 breast cancer xenografts and reduces the frequency of acquired anti-oestrogen resistance

SRD Johnston[1,2], S Riddler[3], BP Haynes[4], R A'Hern[2], IE Smith[2], M Jarman[4] and M Dowsett[1]

Departments of [1]Academic Biochemistry and [2]Medicine, Royal Marsden Hospital, Fulham Road, London SW3 6JJ; [3]Biological Services Unit and [4]Cancer Research Campaign Centre for Cancer Therapeutics, Institute of Cancer Research, Sutton, Surrey SM2 5NG, UK

**Summary** The effect of idoxifene, a novel anti-oestrogen with less agonist activity than tamoxifen, was compared with that of tamoxifen on the growth of hormone-dependent MCF-7 breast cancer xenografts. Forty tumours were established with oestradiol support in ovariectomized athymic mice, allowed to grow to a median volume of 420 mm³ and then continued with oestradiol, no support, tamoxifen or idoxifene delivered by 1.5-cm silastic capsule. Tumour regression occurred with both anti-oestrogens, although maximum regression was observed following oestradiol withdrawal alone. While prolonged anti-oestrogen therapy was associated with static growth, tumour volumes were significantly lower with idoxifene ($P$=0.01). After 6 months, 0/10 idoxifene-treated tumours developed acquired resistance compared with 3/10 tumours treated with tamoxifen. In separate experiments, 94 animals were treated initially with oestradiol, tamoxifen, idoxifene or placebo following implantation with 1-mm³ pieces of either wild-type (WT) or tamoxifen-resistant (TR) MCF-7 tumour. After 4 months, only 1/11 WT tumours became established with idoxifene compared with 4/11 with tamoxifen, 8/12 with oestradiol and 0/12 with placebo. Likewise, fewer TR tumours were supported by idoxifene (3/12) than by tamoxifen (8/12) or oestrogen (11/12). These data indicate that, compared with tamoxifen, idoxifene shows reduced growth support of MCF-7 xenografts and may share only partial cross-resistance. Furthermore, the development of acquired anti-oestrogen resistance may be reduced during long-term idoxifene therapy. The drug's reduced agonist activity may, in part, explain these observations and indicate a preferable biochemical profile for breast cancer treatment.

**Keywords:** breast cancer; idoxifene; tamoxifen; acquired resistance

The anti-oestrogen tamoxifen is established as first-line endocrine therapy for women with breast cancer. In advanced breast cancer, it is most effective in oestrogen receptor (ER)-positive tumours (McGuire, 1978). However, most tumours that respond eventually develop acquired resistance and start to regrow. MCF-7 cells are an ER-positive hormone-dependent human breast cancer cell line, and an animal model using MCF-7 xenografts in athymic mice has been developed by several groups to investigate the phenomenon of acquired anti-oestrogen resistance (Osborne et al, 1985; Gottardis et al, 1988). It has been demonstrated that resistant tumours often become growth dependent on tamoxifen and can be stimulated by the drug in a dose-dependent manner (Gottardis and Jordan, 1988). This growth can be reversed by tamoxifen withdrawal or inhibited by the 'pure' non-steroidal anti-oestrogen ICI 164,384 (Gottardis et al, 1989). It has been suggested that the partial agonist activity of tamoxifen or its metabolites may be responsible for the acquisition of tamoxifen-stimulated growth.

Idoxifene is a novel anti-oestrogen that is structurally related to tamoxifen (Figure 1) (McCague, 1986). Analogues of tamoxifen that include an iodine atom at position 4 have been found to have increased affinity for ER (McCague et al, 1989). Such compounds cannot undergo glucuronidation via 4-hydroxylation, which probably aids the excretion of tamoxifen (McCague et al, 1990*a*), and, unlike *trans*-4-hydroxytamoxifen, they cannot isomerize to the *cis* isomer, which has much weaker anti-oestrogenic properties for tamoxifen, while retaining partial agonist activity (Murphy et al, 1990). In addition, substitution of the dimethylamino group on the side-chain of tamoxifen by the pyrrolidine ring prevents conversion by the liver to desmethyl and didesmethyl metabolites, which are the predominant circulating metabolites of tamoxifen found in humans (Daniel et al, 1981; Jordan et al, 1983). Studies have confirmed that these structural modifications result in a compound that is metabolically more stable than tamoxifen (McCague et al, 1990*b*; Haynes et al, 1991). Idoxifene was more effective than tamoxifen at inhibiting MCF-7 cell growth and rat mammary tumour growth (Chander et al, 1991). Furthermore, observations that idoxifene has reduced agonist activity in the immature rat uterotrophic assay compared with tamoxifen (Chander et al, 1991) suggest that the drug may have a preferable biochemical profile for clinical use, and could be an effective anti-oestrogen in circumstances in which tamoxifen's agonist activity is predominant.

We established a xenograft model to investigate the growth-suppressive activity of idoxifene in acquired tamoxifen-resistant human breast cancer (MCF-7 cells). In particular, we wished to compare in established hormone-dependent xenografts the growth inhibition achieved with idoxifene with that observed with tamoxifen treatment or oestradiol withdrawal. In view of its reported lower agonist activity, we wished to determine whether long-term administration of idoxifene would reduce or delay the emergence of acquired resistance, and whether tamoxifen-resistant tumours would remain sensitive to idoxifene in cross-resistance experiments.

*Received 19 August 1996*
*Revised 18 October 1996*
*Accepted 21 October 1996*

Correspondence to: SRD Johnston, Department of Academic Biochemistry, Royal Marsden Hospital, Fulham Road, London SW3 6JJ, UK

**Figure 1** Chemical structures of the anti-oestrogens tamoxifen and idoxifene

### MATERIALS AND METHODS

### Chemicals

The two anti-oestrogens used in this study were tamoxifen {{Z-*trans*-1-{4-[2-(dimethylamino)ethoxy]phenyl}-1,2-diphenyl-1-butene}} from Sigma Chemical Co., Poole, UK, and idoxifene {E-*trans*-1-(4-iodophenyl)-1-[4-(2-pyrrolidinoethoxy) phenyl]-2-phenyl-1-butene} (McCague, 1986) synthesized at The Cancer Research Campaign Centre for Cancer Therapeutics, Institute of Cancer Research, Sutton, UK.

### Drug delivery system

Slow-release silastic capsules of tamoxifen and idoxifene were made according to previously published methods (Gottardis et al, 1989). Briefly, these were formed by plugging one end of a 1.5-cm length of medical-grade silastic tubing (0.078 inch internal diameter by 0.125 inch outside diameter; Dow Corning, Midland, MI, USA) with silastic 382 medical-grade adhesive. After drying, these were filled with either tamoxifen free base or the crystalline form of idoxifene. Based on the relative molecular weight of each drug (tamoxifen, 371.3; idoxifene, 497.4), an estimated molar equivalent amount of each drug was put into each capsule (20 mg of tamoxifen and 26 mg of idoxifene per capsule), and the capsule was sealed by plugging the open end with adhesive. All capsules were sterilized by γ-irradiation (200 Gy) before subcutaneous implantation under general anaesthetic on the left dorsal paraspinal area.

### Serum drug levels

In preliminary experiments to establish the serum levels and pharmacological profile of each drug, a total of 24 mice were implanted along the left flank (parallel to the spine) with 1.5-cm silastic capsules containing either tamoxifen or idoxifene. Four mice from each group were sacrificed and bled at 2, 4 and between 6 and 8 weeks. The total serum level of each drug was measured at these time points by high-performance liquid chromatography (HPLC) according to previously published methodology (Johnston et al, 1993). The recovery from the mouse serum for each drug was 97%, and the lower detection limit for the assay was 0.1 ng $ml^{-1}$ for tamoxifen and 0.2 ng $ml^{-1}$ for idoxifene.

### Animals and tumours

MCF-7 xenografts were established from cells that had been growing in culture in RPMI-1640 medium (Life Technologies, Paisley, Strathclyde, UK) supplemented with 10% fetal calf serum (Life Technologies.), 2 mM L-glutamine, 5 U $ml^{-1}$ penicillin, 5 mg $ml^{-1}$ streptomycin and 12.5 ng $ml^{-1}$ amphotericin (Sigma Chemical Co.). Cells were recovered from six 80% confluent 175-$cm^2$ flasks by scraping and were resuspended immediately in 2.5 ml of fresh medium. Approximately $10^7$ cells (0.2 ml) were injected in suspension into the right flank of each of ten ovariectomized athymic nude mice (ICRF nunw mice; Harlan, Oxford, UK). At the same time, each mouse received a 1.7-mg 60-day release 17β-oestrodiol ($E_2$) pellet (Innovative Research of America, Toledo, OH, USA) implanted under the neck skin under general anaesthesia. After 8–12 weeks, oestradiol-dependent wild-type (WT) tumours were established for passage in subsequent experiments. All procedures were approved by the Institute of Cancer Research ethics committee.

### Growth inhibition of MCF-7 xenografts

Forty mice were implanted with 1-$mm^3$ pieces of WT tumour in the right flank and supported with E2 pellets implanted at the same time under general anaesthesia. Bidimensional tumour diameters were measured by caliper at weekly intervals, and tumour volume in cubic mm was estimated using the formula:

$$\text{Tumour volume} = \frac{(\text{width})^2 \times \text{length}}{2}$$

At week 9, animals were randomly sorted into four groups of ten, which were treated differently: (1) E2 support with 1.7-mg 60-day release pellet continued; (2) $E_2$ support withdrawn by removal of pellet; (3) tamoxifen; or (4) idoxifene delivered by silastic capsule as described above. In the last two groups, $E_2$ support was withdrawn at the same time as the anti-oestrogen capsule was implanted. Tumour measurements were made weekly and change in tumour size for each animal recorded as the percentage of the baseline size reached at week 9. During prolonged treatment, drug capsules were changed every 60 days under general anaesthesia. The experiment was continued for 6 months to determine whether any tumours would develop acquired resistance and start to regrow.

© Cancer Research Campaign 1997

**Table 1** Serum concentrations of tamoxifen and idoxifene achieved 2, 4 and 6 weeks after implantation of 1.5-cm silastic capsules filled with equimolar amounts of tamoxifen and idoxifene

| Duration of therapy (weeks) | *n* | Tamoxifen (ng ml$^{-1}$) | *n* | Idoxifene (ng ml$^{-1}$) |
|---|---|---|---|---|
| 2 | 4 | 34.3 ± 6.5 | 4 | 17.3 ± 1.2 |
| 4 | 3 | 36.7 ± 6.5 | 4 | 23.8 ± 4.2 |
| 6–8 | 4 | 29.5 ± 5.4 | 3 | 25.3 ± 2.2 |

Values are expressed in ng ml$^{-1}$ as means ± s.e. Detection limits were tamoxifen (0.1 ng ml$^{-1}$) and idoxifene (0.2 ng ml$^{-1}$).

**Figure 2** Effect of tamoxifen, idoxifene or oestradiol withdrawal on growth of MCF-7 xenografts in nude mice. All tumours were initially growth supported with oestradiol. At week 9, capsules were changed to either continued oestradiol, tamoxifen, idoxifene or placebo. Tumour volumes were measured weekly and expressed as the percentage baseline volume at 9 weeks (mean ± s.e.)

### Growth support of WT and TR tumours

Another 48 mice were implanted with 1-mm$^3$ pieces of WT tumour in sequential duplicate experiments (24 mice per experiment). In each experiment, four groups of six mice were supported from the time of tumour implantation with either E2 pellet, placebo pellet, tamoxifen capsule or idoxifene capsule for a total of 16 weeks. The pellets or capsules were replaced under general anaesthesia after 8 weeks. The WT tumour take rates (i.e. number of tumours that become established by week 16) in each group and growth rate of any established tumours were recorded.

In parallel experiments, a further 48 mice were implanted with 1-mm$^3$ pieces from two tamoxifen-resistant (TR) tumours that had developed acquired tamoxifen resistance during long-term tamoxifen treatment. The original $E_2$-established WT tumours from these two mice had regressed following tamoxifen therapy, but after 18 and 21 weeks, started to regrow despite continued tamoxifen. In a similar design to the experiments with WT tumour, four groups of six mice (repeated in duplicate) were supported with $E_2$, placebo, tamoxifen or idoxifene for 16 weeks, and the TR tumour take rate and growth rate were recorded.

**Figure 3** Growth rate of three MCF-7 xenografts that regrew during prolonged tamoxifen therapy and developed acquired resistance. Tamoxifen capsules were changed every 8 weeks as indicated by the arrows

### Statistics

Growth rates for individual tumours were calculated assuming an exponential model within individual time segments. The growth rate was calculated as the slope of the line of log (volume) plotted against time. Comparisons in growth rate were performed using the Kruskal–Wallis one-way analysis of variance for the three treatment groups (tamoxifen, idoxifene and placebo), with the Mann–Whitney test for two samples using a multiple comparison corrected *P*-value of 0.017 (i.e. 0.05/3).

### RESULTS

### Serum tamoxifen and idoxifene concentrations

In preliminary experiments with 24 mice, the serum concentrations of tamoxifen and idoxifene obtained using the slow-release silastic capsules were measured after 2, 4 and 6 weeks. These data are shown in Table 1. By 4 and 6 weeks when steady state would be expected, the mean concentration of tamoxifen and idoxifene was 35.3 ± 4.3 ng ml$^{-1}$ and 28.4 ± 2.2 ng ml$^{-1}$, respectively, which is equivalent to 95 nM for tamoxifen and 57 nM for idoxifene.

### Growth inhibition of MCF-7 xenografts

Tumour size obtained after initial oestradiol support varied markedly with the smallest tumour measuring 78 mm$^3$ and the largest 2790 mm$^3$. However, the median tumour size and ranges in each of the four groups of ten mice were similar (413, 342, 434 and 366 mm$^3$) such that valid comparisons in growth inhibition could be made between groups.

Tumours treated with further E2 support continued to grow at a steady rate. Tumours in which E2 support was withdrawn regressed in size such that by week 26 the mean tumour size was

 © Cancer Research Campaign 1997

**Figure 4** Effect of oestrogen, tamoxifen and idoxifene on the growth support of tamoxifen-resistant (TR) MCF-7 xenografts. Tumour implants from an acquired tamoxifen-resistant tumour (1 $mm^3$ size) were implanted on day 0 into 48 mice that were then randomly allocated to receive oestradiol, tamoxifen, idoxifene or placebo by silastic capsule (capsules replaced again after 8 weeks). The weekly mean tumour volumes are displayed (± s.e.) for the tumours that became established (take-rate indicated in brackets)

12% of baseline (Figure 2) and 3/10 tumours had regressed completely. In mice treated with tamoxifen, tumour growth continued initially and peaked at 112% at week 10. Thereafter, tamoxifen induced tumour regression, although this was significantly less than that observed following E2 withdrawal ($P$=0.0003). Idoxifene induced immediate tumour regression that was significantly greater than that in the tamoxifen-treated group ($P$=0.01) (Figure 2).

During prolonged anti-oestrogen therapy, tumour volumes in mice treated with tamoxifen remained static and 3/10 tumours started to regrow after an interval of 15, 18 and 21 weeks of therapy respectively. The growth of these individual tumours that developed acquired tamoxifen resistance is shown in Figure 3. In contrast, no tumours treated with idoxifene started to regrow during this same time period.

### Growth support of wild-type (WT) and tamoxifen-resistant (TR) tumours

Two of the 96 animals (one tamoxifen WT and one idoxifene WT) died shortly after tumour passage and capsule implantation (necropsies were not performed). The remaining 94 animals were observed for 16 weeks. WT tumour growth was dependent on E2 support, as 0/12 tumours grew with placebo compared with 8/12 treated with E2, these latter tumours arising after a median of 3 weeks. With tamoxifen, 4/11 tumours took after a median of 9 weeks, whereas only 1/11 WT tumours treated with idoxifene became established (and then only at week 15).

The growth of TR tumours was also dependent on endocrine support to become established; 11/12 E2-supported tumours took and grew at rates similar to those observed with WT tumours, whereas 0/12 tumours took when treated with placebo. More TR tumours were supported by tamoxifen than by idoxifene (8/12 vs 3/12), although this was not statistically significant ($P$=0.0995, Fisher's exact test, two-tail). No difference was observed between the TR tumour growth rates in idoxifene and tamoxifen-treated animals, although the median time to tumour take was longer with idoxifene (9 weeks vs 5 weeks). Growth rates with both anti-oestrogens were slower than with oestradiol (Figure 4).

### DISCUSSION

MCF-7 xenografts established in ovariectomized nude mice and treated with long-term tamoxifen provide a model for investigating acquired anti-oestrogen resistance in breast cancer. Previous studies have established that, while tamoxifen will cause partial regression of established xenografts, continued administration is associated with static growth and stable tumour volumes (Osborne et al, 1985; Gottardis et al, 1988). Following prolonged therapy, resistance eventually develops in this model and tumours regrow despite continued tamoxifen (Gottardis and Jordan, 1988). Subsequent studies have shown these tumours to be growth-stimulated by tamoxifen in a dose-dependent manner and that growth can be reduced by withdrawal of tamoxifen (Gottardis et al, 1989). In cross-resistance experiments, the pure anti-oestrogen, ICI 164,384, can inhibit tamoxifen-stimulated growth of these resistant tumours (Gottardis et al, 1989), which implies a mechanism for acquired resistance that is specific to tamoxifen. This mechanism would explain the clinical observation that more than 50% of patients with advanced breast cancer, who previously responded to tamoxifen before developing acquired resistance, will respond to further endocrine therapies with either pure anti-oestrogens (Howell et al, 1995) or aromatase inhibitors (Dowsett et al, 1995).

Acquired tamoxifen resistance may be associated with the agonist properties of the drug and/or its metabolites (Howell et al, 1990). Several of the known metabolites have more agonist than antagonist effects. These include compounds formed following metabolism of the dimethylamino side-chain (i.e. the monophenol metabolite E and the bisphenol) (Lyman and Jordan, 1985). Although the *cis* isomer of 4-hydroxytamoxifen is probably as oestrogenic as the *trans* isomer, it is a much weaker antagonist (McCague et al, 1990*a*). It has been suggested that a relative increase within the tumour of more oestrogenic metabolites of tamoxifen could stimulate growth (Osborne et al, 1991; Wiebe et al, 1992). Analogues of tamoxifen in which the formation of these metabolites is prevented or reduced may prove more effective anti-oestrogens, and theoretically could delay the onset of any acquired resistance that was caused by stimulation by agonist metabolites.

Idoxifene is a structural analogue of tamoxifen that is metabolically more stable than tamoxifen. In vitro studies, using isolated rat hepatocytes, demonstrated that idoxifene was metabolized approximately three times more slowly than tamoxifen (Haynes et al, 1991). In vivo idoxifene was shown to have a significantly longer terminal half-life than tamoxifen in the rat (Haynes et al, 1991). In a recent phase I study in women with advanced breast cancer (Coombes et al, 1995), the terminal half-life of idoxifene was 3 weeks compared with a known half-life for tamoxifen of 7 days (DeVos et al, 1992). In addition, the antagonist/agonist profile for idoxifene appears favourable to that for tamoxifen. Idoxifene has a greater relative binding affinity (RBA) for the

© Cancer Research Campaign 1997

oestrogen receptor compared with tamoxifen (tamoxifen, 5; idoxifene, 12.5; oestradiol, 100) and is 1.5-fold more effective than tamoxifen at inhibiting oestrogen-induced MCF-7 cell growth (Chander et al, 1991). Idoxifene caused a greater percentage of tumour regression in the hormone-dependent NMU-induced rat mammary tumour model than tamoxifen (92% vs 75%). In uterotrophic studies in immature rats and mice, idoxifene had reduced agonist activity compared with tamoxifen in doses up to 10 mg $kg^{-1}$ (Chander et al, 1991). Overall, these data suggest that, compared with tamoxifen, idoxifene is metabolically more stable, has reduced agonist activity and inhibits hormone-dependent tumour growth more effectively.

Our data demonstrate greater tumour inhibition by idoxifene of MCF-7 xenografts compared with tamoxifen in vivo. Equimolar amounts of each drug were delivered by slow-release silastic capsule and resulted in relatively similar serum concentrations that remained stable during the 8 weeks each capsule was implanted. The slightly lower mean levels (57 nM vs 95 nM) could have resulted from variation in the release characteristics through silastic as a consequence of the idoxifene's different chemical structure. Unlike oestrogen withdrawal in this model in which tumour size diminished to 12% of baseline after 6 months, both tamoxifen and idoxifene maintained static tumour volumes following an initial period of tumour regression (Figure 2). The biological basis for these stable volumes remains unclear. Classical anti-oestrogens are thought to be cytostatic in action inhibiting cell cycle progression through $G_1$ (Sutherland et al, 1983), and initial reduction in tumour volume may represent increased cell loss and/or reduced cell proliferation owing to antagonism of the mitogenic signal. The advantage of this MCF-7 animal model, namely anti-oestrogen therapy in oophorectomized mice after removal of the oestradiol pellet, is that it allows the inherent agonist activity of the two drugs to be evaluated in the absence of endogeneous or exogenous oestradiol. The significant difference in the baseline level at which tumour size was maintained between idoxifene and tamoxifen during long-term anti-oestrogen therapy could reflect the different agonist profiles of the two agents.

The acquisition of resistance following prolonged therapy was seen only in tamoxifen-treated tumours. None of the idoxifene-treated tumours regrew during the 6-month experiment, although it is possible that with more prolonged therapy idoxifene-resistant tumours would have developed. Similar experiments that compared the effects of the pure anti-oestrogen, ICI 182,780, with those of tamoxifen in the same MCF-7 xenograft model demonstrated that the pure anti-oestrogen suppressed tumour growth for twice as long as treatment with tamoxifen (Osborne et al, 1995). Eventually, most tumours became resistant to ICI 182,780, although these experiments were conducted for much longer (11 months) compared with the idoxifene studies. However, both studies imply that more effective oestrogen antagonism using drugs with reduced agonist activity may provide not only greater inhibition of tumour growth than tamoxifen, but may delay the onset of acquired resistance. Clearly, such a property would be highly advantageous for a novel endocrine agent, if this were translated in the clinic into prolonged time to disease progression.

Experiments in which tumour implants are growth supported from the outset with either oestrogen or anti-oestrogen allow a comparison of the tumorigenic potential of each drug to be made. Fewer wild-type MCF-7 tumours were growth supported by idoxifene compared with tamoxifen, with a longer time to tumour formation and reduction in tumour take-rate. Similar observations have been reported with wild-type MCF-7 xenografts treated with the pure anti-oestrogen, ICI 182,780, compared with tamoxifen (Osborne et al 1995). These data support those from experiments that study growth inhibition of established MCF-7 tumours and suggest that anti-oestrogens with less agonist activity are less likely to support tumour growth.

Tamoxifen-resistant (TR) tumours were growth supported by tamoxifen, although this phenotype clearly remained hormone dependent as illustrated by the lack of tumours that developed in the absence of any exogenous hormone. Idoxifene-supported tumours were less frequent and developed later after a median of 15 weeks compared with 9 weeks for tamoxifen. Although there was no statistical difference in the growth rates of idoxifene- and tamoxifen-supported TR tumours, the latter were still growing actively at 16 weeks when the experiment was terminated (Figure 4). The emergence of tumours later that grow more slowly may represent greater sensitivity of TR tumours to idoxifene. This could be interpreted as a lack of partial cross-resistance between the two anti-oestrogens, although the hormone dependence of these idoxifene-supported tumours was not examined in further serial transplant experiments. However, when ICI 182,780-resistant tumours developed after 11 months and were transplanted into new mice, they were noted to have become completely endocrine independent and grew in the absence of oestradiol (Osborne et al, 1995).

These data imply that novel anti-oestrogens, such as idoxifene or ICI 182,780, may inhibit the growth of tamoxifen-resistant tumours more effectively, a feature which could be related to the drug's reduced agonist activity. If the formation of oestrogenic metabolites were a significant mechanism for tamoxifen relapse, then structural analogues in which their formation is prevented or reduced could be more effective anti-oestrogens. However, recent data from two separate groups have shown that using fixed-ring derivatives of tamoxifen in which isomerization is inhibited (thus preventing formation of the *cis* isomer of 4-hydroxytamoxifen), growth stimulation of resistant tumours occurred to the same extent as with tamoxifen itself (Wolf et al, 1993; Osborne et al, 1994). Furthermore, analogues, such as deoxytamoxifen (in which cleavage of the dimethylamino side-chain is impaired, thus reducing formation of the oestrogenic metabolite E or bisphenol), nafoxidine and toremifene all stimulated tumour growth (Osborne et al, 1994). Thus, the mechansism for tamoxifen-stimulated growth in this model remains unclear. Nonetheless, these data imply that the contribution of oestrogenic metabolites of tamoxifen in stimulating the growth of established acquired tamoxifen-resistant tumours is probably low. However, it remains possible that structural analogues of tamoxifen with substantially less agonist activity are more effective at inhibiting the growth of hormone-sensitive tumours, which, compared with tamoxifen, may result in the delayed onset of acquired resistance. If this translated in the clinical setting into prolonged disease control in the primary or adjuvant setting, this would represent a significant advantage for idoxifene over tamoxifen, currently the first-line endocrine therapy for breast cancer.

### ACKNOWLEDGEMENTS

This work was funded by British Technology Group, Ltd. SRDJ is a Cancer Research Campaign Clinical Research Training Fellow.

 © Cancer Research Campaign 1997

**REFERENCES**

Chander SK, McCague R, Luqmani Y, Newton C, Dowsett M, Jarman M and Coombes RC (1991) Pyrrolidino-4-iodotamoxifen and 4-iodotamoxifen, new analoques of the antioestrogen tamoxifen for the treatment of breast cancer. *Cancer Res* **51**: 5851–5858

Coombes RC, Haynes BP, Dowsett M, Qugley M, English J, Judson IR, Griggs LJ, Potter GA, McCague R and Jarman M (1995) Idoxifene: report of a phase I study in patients with metastatic breast cancer. *Cancer Res* **55**: 1070–1074

Daniel P, Gaskell SJ, Bishop H, Campbell C and Robertson RI (1981) Determination of tamoxifen and biologically active metabolites in human breast tumours and plasma. *Eur J Cancer Clin Oncol* **17**: 1183–1189

DeVos D, Slee PHTJ, Stevenson D and Briggs LJ (1992) Serum elimination half-life of tamoxifen and its metabolities in patients with advanced breast cancer. *Cancer Chemother Pharamacol* **31**: 76–78

Dowsett M, Johnston SRD, Iveson TJ and Smith IE (1995) Response to pure antioestrogen (ICI 182, 780) in tamoxifen-resistant breast cancer. *Lancet* **345**: 525

Gottardis M and Jordan VC (1988) Development of tamoxifen-stimulated growth of MCF-7 tumors in athymic mice after long-term antioestrogen administration. *Cancer Res* **48**: 5183–5187

Gottardis MM, Robinson SP and Jordan VC (1988) Estradiol-stimulated growth of MCF-7 tumors implanted in athymic mice: a model to study the tumoristatic action of tamoxifen. *J Steroid Biochem* **30**: 311–314

Gottardis MM, Jiang S-Y, Jeng M-H and Jordan VC (1989) Inhibition of tamoxifen-stimulated growth of an MCF-7 tumor variant in athymic mice by novel steroidal antiestrogens. *Cancer Res* **49**: 4090–4093

Haynes BP, Parr IB, Griggs LJ and Jarman M (1991) Metabolism and pharmacokinetics of pyrrolidino-4-iodotamoxifen in the rat. *Breast Cancer Res Treat* **19**: 174 (A72)

Howell A, De Friend D, Robertson J, Blamey R and Walton P (1995) Response to the pure antioestrogen ICI 182,780 in tamoxifen resistant breast cancer. *Lancet* **345**: 29–30

Howell A, Dodwell DJ, Laidlaw I, Anderson H and Anderson E (1990) Tamoxifen as an agonist for metastatic breast cancer. In *Endocrine Therapy of Breast Cancer*, Goldhirsch A (ed.), pp. 49–58 Springer: New York

Johnston SRD, Haynes BP, Sacks NPM, McKinna JA, Griggs LJ, Jarman M, Baum M, Smith IE and Dowsett M (1993) Effect of oestrogen receptor status and time on the intra-tumoural accumulation of tamoxifen and N-desmethyltamoxifen following short-term therapy in human breast cancer. *Breast Cancer Res Treat* **28**: 241–250

Jordan VC, Bain RR, Brown RR, Gosden B and Santos MA (1983) Determination and pharmacology of a new hydroxylated metabolite of tamoxifen observed in patient sera during therapy for advanced breast cancer. *Cancer Res* **43**: 1446–1450

Lyman SD and Jordan VC (1985) Metabolism of tamoxifen and its uterotrophic activity. *Biochem Pharmacol* **34**: 2787–2794

McCague R (1986) Halogenated tamoxifens. British Patent Application No 8621908

McCague R, Leclercq G, Legros N, Goodman J, Blackburn GM, Jarman M, et al. (1989) Derivatives of tamoxifen; dependence of antioestrogenicity on the 4-substituent. *J Med Chem* **32**: 2527–2533

McCague R, Parr IB, Leclercq G, Leung O-T and Jarman M (1990*a*) Metabolism of tamoxifen by isolated rat hepatocytes; identification of the glucuronide of 4-hydroxytamoxifen. *Biochem Pharmacol* **39**: 1459–1465

McCague R, Parr IB and Haynes BP (1990*b*) Metabolism of 4-iodo derivative of tamoxifen by isolated rat hepatocytes. *Biochem Pharmacol* **40**: 2277–2283

McGuire WL (1978) Hormone receptors; their role in predicting prognosis and response to endocrine therapy. *Semin Oncol* **5**: 428–433

Murphy CS, Langan FS, McCague R and Jordan VC (1990) Structure–function relationships of hydroxylated metabolites of tamoxifen that control the proliferation of estrogen-responsive T47D breast cancer cells *in vitro*. *Mol Pharmacol* **38**: 737–743

Osborne CK, Hobbs K and Clark GM (1985) Effect of estrogens and antiestrogens on growth of human breast cancer cells in athymic nude mice. *Cancer Res* **45**: 584–590

Osborne CK, Coronado E, Allred DC, Wiebe V and DeGregorio M (1991) Acquired tamoxifen resistance: correlation with reduced breast tumor levels of tamoxifen and isomerisation of trans-4-hydroxytamoxifen. *J Natl Cancer Inst* **83**: 1477–1482

Osborne CK, Jarman M, McCague R, Coronado EB, Hilsenbeck SG and Wakeling AE (1994) The importance of tamoxifen metabolism in tamoxifen-stimulated breast tumor growth. *Cancer Chemother Pharmacol* **34**: 89–95

Osborne CK, Coronado-Heinsohn EB, Hilsenbeck SG, McCue BL, Wakeling AE, McClelland RA, Manning DL and Nicholson RI (1995) Comparison of the effects of a pure steroidal antiestrogen with those of tamoxifen in a model of human breast cancer. *J Natl Cancer Inst* **87**: 746–750

Sutherland RL, Green MD, Hall RD, Reddell RR and Taylor IW (1983) Tamoxifen induces accumulation of MCF-7 human mammary carcinoma cells in the G0/G1 phase of the cell cycle. *Eur J Cancer Clin Oncol* **19**: 615–621

Wiebe VJ, Osborne CK, McGuire WL and DeGregorio MW (1992) Identification of estrogenic tamoxifen metabolite(s) in tamoxifen-resistant human breast tumors. *J Clin Oncol* **10**: 990–994

Wolf DM, Langhan-Fahey SM, Parker CJ, McCague R and Jordan VC (1993) Investigation of the mechanism of yamoxifen-stimulated breast tumor growth with nonisomerisable analogues of tamoxifen and metabolites. *J Natl Cancer Inst* **85**: 806–812

© Cancer Research Campaign 1997